# CKDNET, a quality improvement project for prevention and reduction of chronic kidney disease in the Northeast Thailand

**DOI:** 10.1186/s12889-020-09387-w

**Published:** 2020-08-27

**Authors:** Ubon Cha’on, Kanok Wongtrangan, Bandit Thinkhamrop, Sajja Tatiyanupanwong, Chulaporn Limwattananon, Cholatip Pongskul, Thanachai Panaput, Chalongchai Chalermwat, Worachart Lert-itthiporn, Amod Sharma, Sirirat Anutrakulchai, CKDNET group

**Affiliations:** 1 Department of Biochemistry, Faculty of Medicine, Khon Kaen University, Khon Kaen, Thailand; 2 Chronic Kidney Disease Prevention in the Northeast of Thailand (CKDNET), Khon Kaen University, Khon Kaen, Thailand; 3 Data Management and Statistical Analysis Center, Faculty of Public Health, Khon Kaen University, Khon Kaen, Thailand; 4 Chaiyaphum Hospital, Chaiyaphum, Thailand; 5 Faculty of Pharmaceutical Science, Khon Kaen University, Khon Kaen, Thailand; 6 Department of Internal Medicine, Faculty of Medicine, Khon Kaen University, Khon Kaen, Thailand; 7 Khon Kaen Hospital, Khon Kaen, Thailand

**Keywords:** Chronic kidney disease, CKD registry, CKDNET, Interventions, Model care

## Abstract

**Background:**

The incidence of chronic kidney disease (CKD) is high in the Northeast Thailand compared to other parts of the country. Therefore, a broad program applying all levels of care is inevitable. This paper describes the results of the first year trial of the Chronic Kidney Disease Prevention in the Northeast Thailand (CKDNET), a quality improvement project collaboratively established to curb CKD.

**Methods:**

We have covered general population, high risk persons and all stages of CKD patients with expansive strategies such as early screening, effective CKD registry, prevention and CKD comprehensive care models including cost effectiveness analysis.

**Results:**

The preliminary results from CKD screening in general population of two rural sub-districts show that 26.8% of the screened population has CKD and 28.9% of CKD patients are of unknown etiology. We have established the CKD registry that has enlisted a total of 10.4 million individuals till date, of which 0.13 million are confirmed to have CKD. Pamphlets, posters, brochures and other media of 94 different types in the total number of 478,450 has been distributed for CKD education and awareness at the community level. A CKD guideline that suits for local situation has been formulated to deal the problem effectively and improve care. Moreover, our multidisciplinary intervention and self-management supports were effective in improving glomerular filtration rate (49.57 versus 46.23 ml/min/1.73 m^2^; *p* < 0.05), blood pressure (129.6/76.1 versus 135.8/83.6 mmHg) and quality of life of CKD patients included in the program compared to those of the patients under conventional care. The cost effectiveness analysis revealed that lifetime cost for the comprehensive health services under the CKDNET program was 486,898 Baht compared to that of the usual care of 479,386 Baht, resulting in an incremental-cost effectiveness ratio of 18,702 Baht per quality-adjusted life years gained.

**Conclusion:**

CKDNET, a quality improvement project of the holistic approach is currently applying to the population in the Northeast Thailand which will facilitate curtailing of CKD burden in the region.

## Background

Chronic Kidney Disease (CKD) is one of the major public health problems across the world. According to the Global Burden of Disease study 2015, kidney disease was the 12th most common cause of death, accounting for 1.1 million deaths per year worldwide [1]. CKD has affected about 11–13% of the population worldwide [2] and about 91% of them are unaware of getting it [3]. Diabetes mellitus and hypertension are the major associated risk factors for the development of CKD [4, 5], which may turn CKD into a final and fatal condition of the end stage renal disease (ESRD). Furthermore, CKD of unknown origin (CKDu) is alarming the experts in various places of the world [6–8].

In 2009, the Thai- Screening and Early Evaluation of Kidney Disease (SEEK) study group reported that the prevalence of CKD in Thailand is 17.5% with stages I, II, III and IV 3.3, 5.6, 7.5 and 1.1%, respectively [9]. The highest prevalence was noted in Bangkok (23.9%), followed by the Northeast (22.2%) and North (20.4%) regions [9]. In the Northeast Thailand, not only the incidence of kidney stone, but also of probable kidney diseases are high compared to other regions [10, 11]. Moreover, awareness of the CKD among the Thai population is extremely low [9]. This is vital because earlier detection of CKD allows timely intervention, potentially slower the progression of disease, and decrease mortality [12, 13]. Based on the high prevalence of CKD, the policy to mitigate CKD burden was developed in Thailand by setting up CKD clinics in various hospitals. However, we have explored and found the barriers of CKD reduction including;- (i) lack of national registry of CKD, (ii) insufficient number of trained case-manager nurses, (iii) unawareness of risk factors or unable to change behavior of people, (iv) no early CKD detection measures and (v) inadequate supports from society, community and policy makers.

The Northeast Thailand covers 20 provinces in the area of about 168,000 km^2^ with cities, smaller towns and rural settings, and has a population of about 22 million. Agriculture is the largest sector of economy and the majority of the population relay on government health services as such all districts have a hospital and all sub-districts have clinics providing primary health care. Comprehending the CKD burden, socio-economic factors and health facilities in the region, a quality improvement project, “Chronic Kidney Disease Prevention in the Northeast Thailand” (CKDNET), has been established for activities such as screening, surveillance, diagnosis, treatment, awareness and management of kidney diseases. The main objectives of the CKDNET include i) revealing CKD burden, associated risk factors and prevention; ii) developing CKD registry system; iii) providing comprehensive care; and iv) developing a cost-effective model care. These goals of the CKDNET will be achieved through various sub-projects as listed in Table 1. In other words, the CKDNET is a quality improvement project by holistic approach to promote best practice and outcomes.

**Table 1:** List of the sub-projects under the CKDNET

	Sub-Projects
1	Prevention and reduction of chronic kidney disease in urban areas
2	Kidney disease prevention and reduction program in rural communities
3	Development and maintenance of information technology systems for CKD
4	Development of the comprehensive care in CKD
5	Cost-effectiveness analysis of viewing programs in patients with CKD
6	Innovative engineering projects in kidney disease
7	Impact of climate change and global warming in chronic non-communicable diseases

This article outlines strategies and activities of the CKDNET including the preliminary findings from CKD screening program in general population, surveillance of CKD patients among hospitals in the Northeast Thailand and provide the results of cost effectiveness analysis of our comprehensive care model in comparison to the conventional care. We believe that our findings will provide a direction for further improvement including research, knowledge, funding distribution and capacity in the care of CKD patients.

## Methods/design

### Structure and workflow

**Fig. 1:**
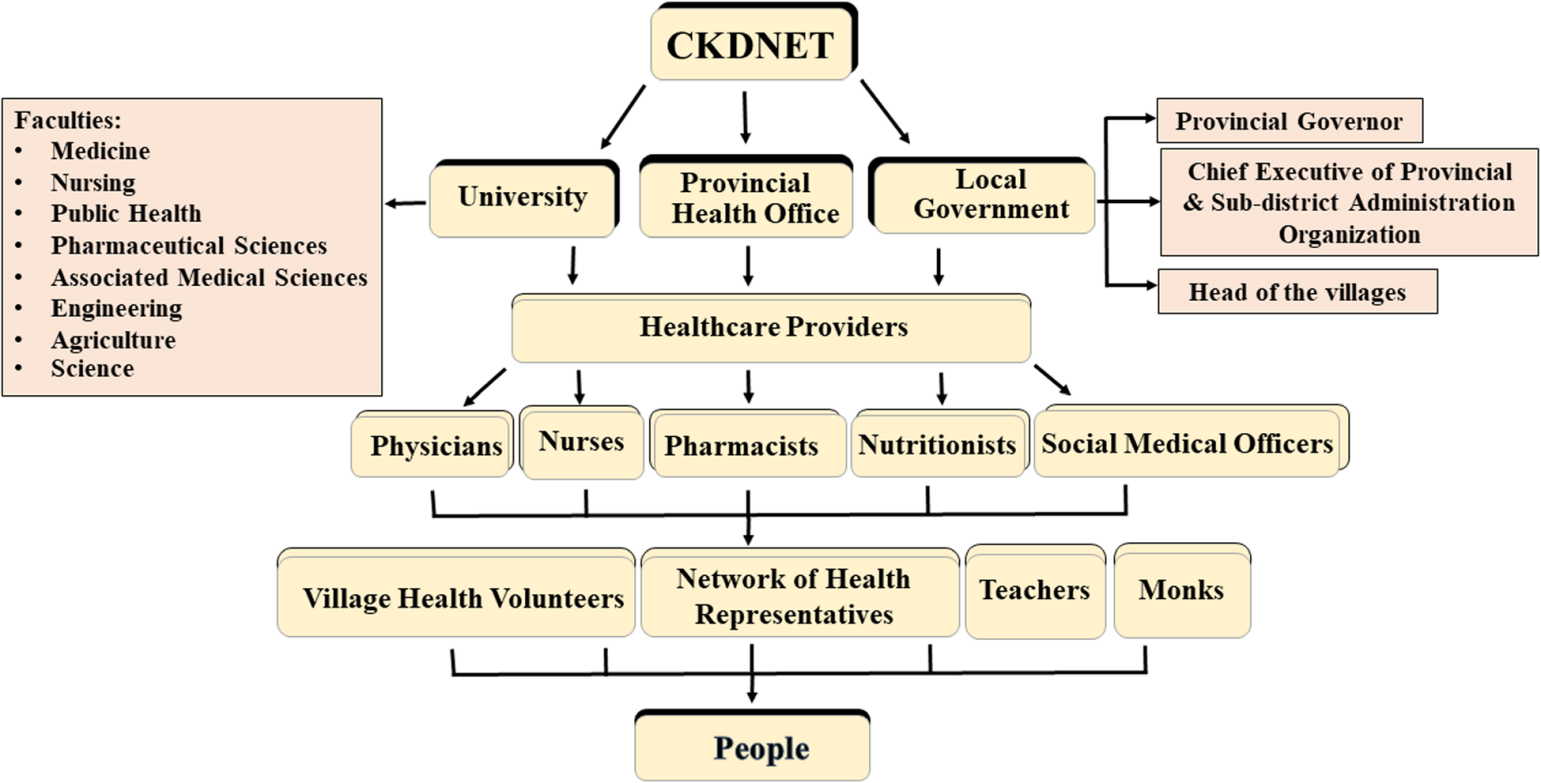
The workflow of CKDNET project. The activities progress through the faculties of Khon Kaen University, local government and provincial health office, followed in next level by physicians, nurses, pharmacists, village health volunteers and others

The CKDNET has been operating in the Faculty of Medicine, Khon Kaen University (KKU), Thailand, in collaboration with public health sectors since 2016. The project was approved by the Khon Kaen University Ethics Committee for Human Research on May 12th, 2017 (HE601166). A multidisciplinary panel of KKU investigators from the Faculties of Medicine, Nursing, Public Health, Pharmaceutical Sciences, Associated Medical Sciences, Science, Engineering, and Agriculture have been participating in this project. Numerous strategies have been applied to deal with the CKD problems by sharing knowledge and co-operations with local governments, health authorities, network of health representatives, village health volunteers and people. The workflow of the CKDNET is illustrated in Fig. 1. All activities of our quality enhancement project are governed by the management committee, and the sub-committees are responsible for specific research projects [14].

### Strategies

**Fig. 2:**
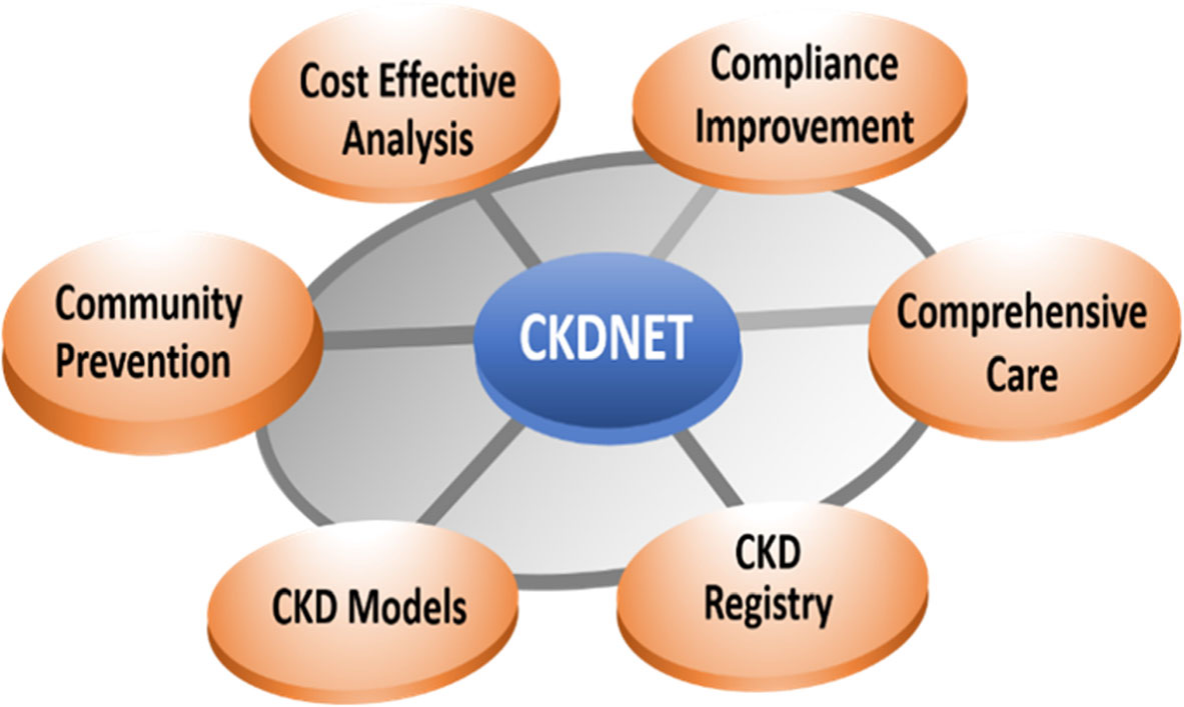
Strategies of CKDNET for its activities (known as 6C)

Various strategies such as epidemiological studies, surveillance, coordinated models of nurses and advanced practitioners, integration with dieticians and social workers, and education programs, has been implicated against CKD around the world [15–18]. Taking these approaches into consideration, the CKDNET has designed and implemented six different strategies as a roadmap for its activities combating with the obstacles of CKD reduction to achieve the objectives covering all levels of population and diseases with cost-effectiveness (Fig. 2).

#### CKD screening in rural areas and identification of related factors

**Fig. 3:**
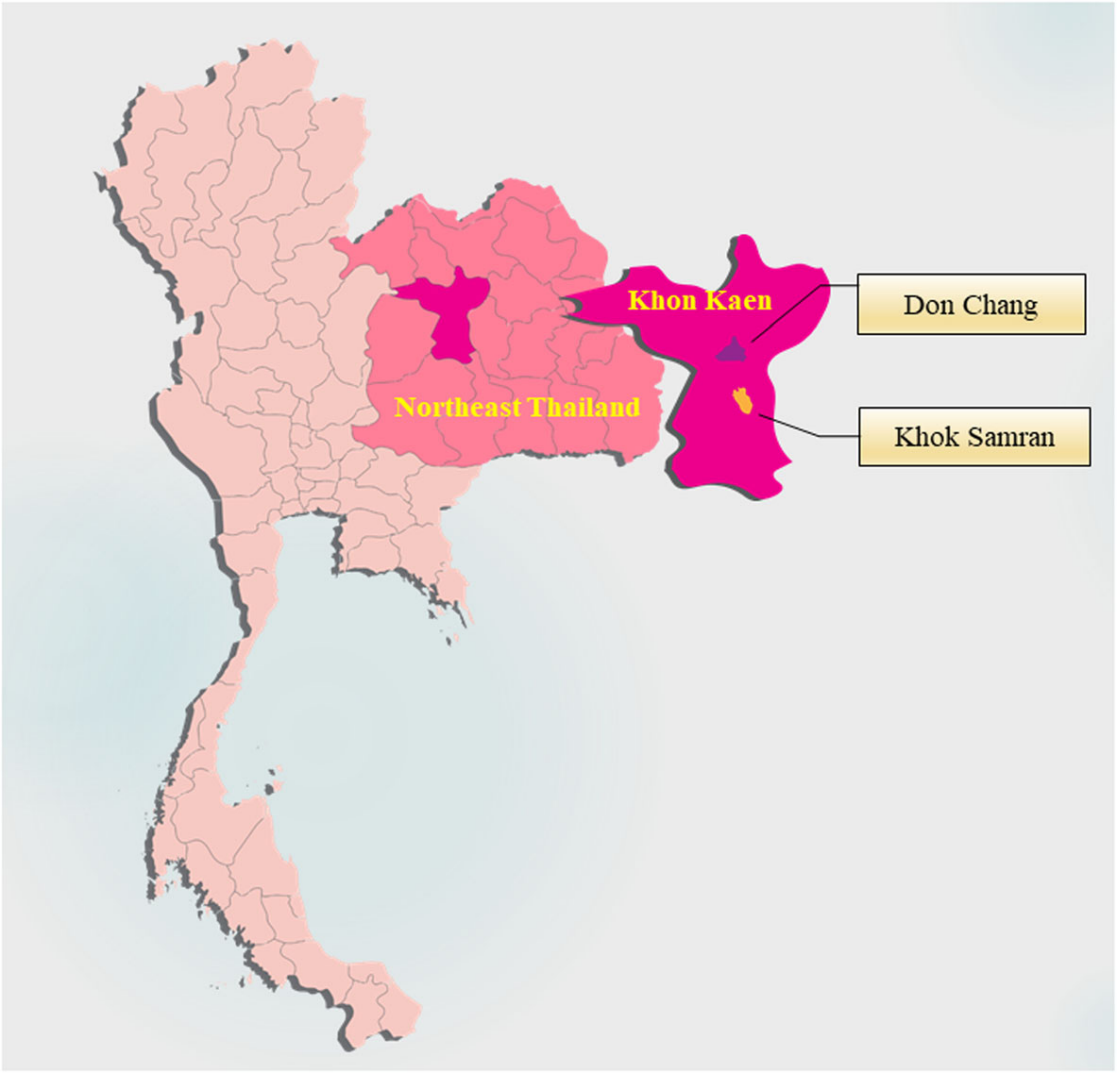
Map of Thailand, the northeastern region and pilot study sites. The figure was created with Adobe Illustrator CC using “locator map of Khon Kaen Province, Thailand” by NordNordWest licensed under CC BY 3.0

The CKD screening program among rural population was started at Don Chang Sub-district of Khon Kaen Province in 2017 and was extended to Khok Samran Sub-district in the same Province in 2018 (Fig. 3). The aim is to identify CKD patients at their early phase. The target villages were selected considering true depiction of the Northeast Thailand population, health status of the villages as well as co-operation from local healthcare units and community leaders. A brief introduction of the program and the aims of the study was delivered to the villagers through the health volunteers, and asked for their consent to participate. Any local inhabitant aged ≥18 years without fever, weight loss > 10% within 3 months and amputation were included in the study.

During the CKD screening process, socio-epidemiological data were collected by interviewing the participants on pre-designed questionnaire. Body weight, height and blood pressure were measured including body fat and water content (using bio-impedance). Arterial blood pressure was measured using automated digital sphygmomanometers after a 15 min rest. All participants were informed to maintain 8-h overnight fasting for blood and urine sample collection. The samples were analysed for serum creatinine, blood glucose and routine urine tests. Urine albumin/creatinine ratio was also calculated for the samples. Moreover, abdominal ultrasonography of the participants was carried out to find out structural abnormalities in the kidneys. CKD was defined according to the guideline of Kidney Disease Improving Global Outcomes *(*KDIGO), indicating presence of kidney damage and/or reduction of estimated glomerular filtration rate (eGFR) < 60 ml/min/1.73 m^2^ for 3 months or longer [19]. The prevalence and risk factors of overall CKD and CKDu were identified upon statistical analysis.

#### CKD surveillance in hospital-based level

**Fig. 4:**
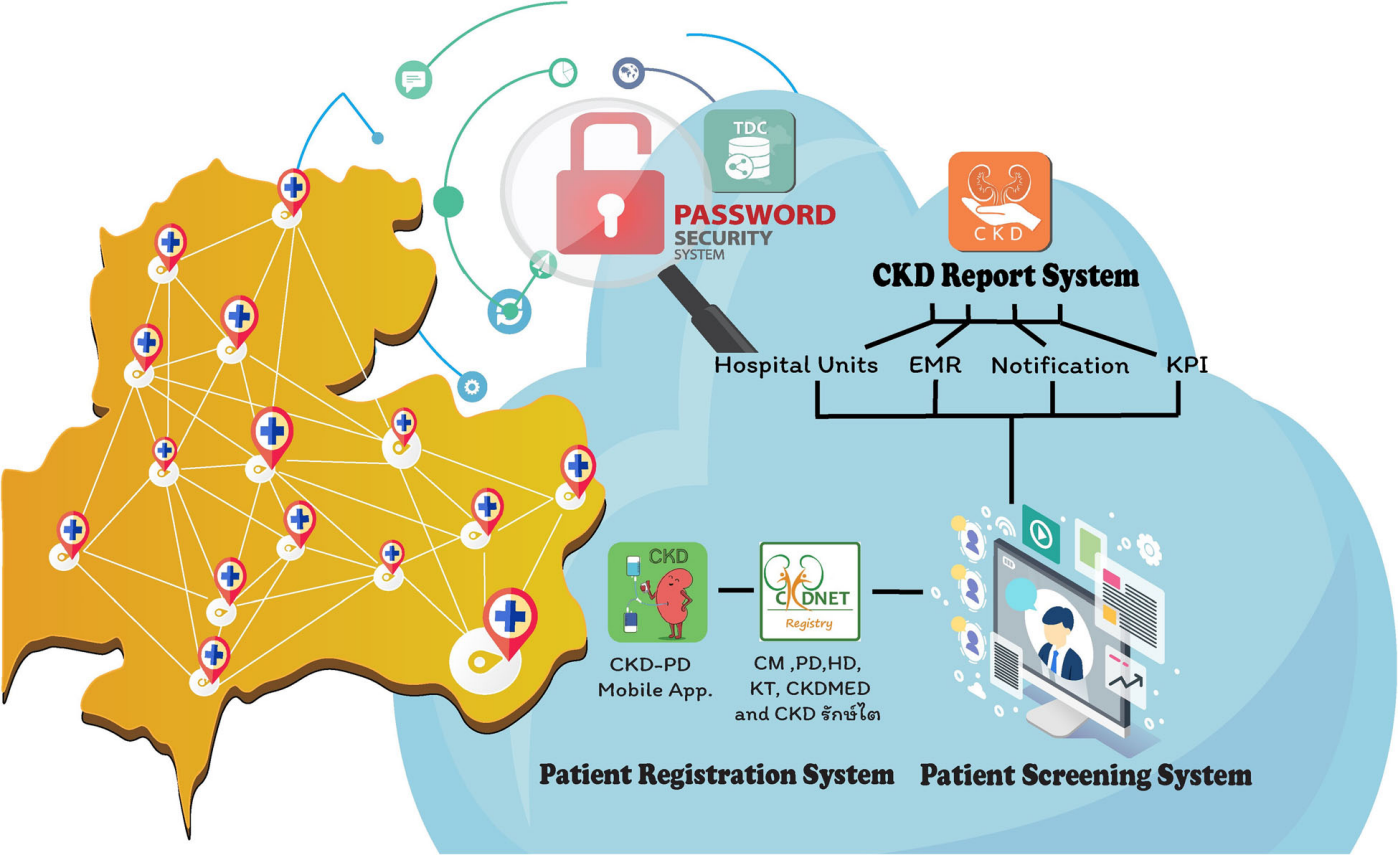
CKD Surveillance components (www.thaicarecloud.org). The surveillance system consists of data encryption technology which is installed at hospitals server to export data to Thai Care Cloud from CKD report system, patient registration system and patient screening system

A web-based application, the CKDNET module has been developed by the Data Management and Statistical Analysis Center (DAMASAC), Faculty of Public Health, KKU. The CKDNET module is a disease registry database as well as a disease surveillance system, working in the Thai health care platform called Thai Care Cloud (TCC) at www.thaicarecloud.org. To use the CKDNET module, the Information Technology (IT) personnel of the participating hospital needs to install a tiny Thai Database Connector (TDC) in the server of the hospital information system (HIS). The TDC breaks the barriers among HIS vendors by providing a mapping tool for IT personnel to enable the different HIS to export a unified data set to the TCC automatically in the real time manner. While doing so, patients’ identification data such as name, house number, village number and others are encrypted, and the system creates the platform of EMR records for CKD patients and further processes and analyses information before presenting a real time summarized report. Moreover, the CKDNET has also introduced an innovative approach to track the kidney function and related health through mobile applications (CKD-PD app). When users are permitted, the personal data gets enlist in the CKDNET registry and local physicians or nurses can communicate with them. A brief overview of the surveillance components is shown in Fig. 4.

#### Community-based CKD prevention

We have adopted both primary and secondary prevention strategies. One of the primary CKD prevention plans applied is a salt intake reduction program. It involves the measurement of salt content in food prepared at restaurants and canteens, followed by a salt intake reduction campaign. The salt reduction campaign for school teachers and students has been implemented with the assumption that they can deliver awareness more intensely. Moreover, promotion of CKD awareness through various prints, social and electronic media on lifestyle modification such as diet, regular physical examination, medication, exercise and others constitutes our foremost activity. Another decisive strategy adopted is secondary prevention by creating awareness among primary health care providers and health volunteers to identify those at risk for CKD in the community; for example, hypertensive and diabetic patients in order to initiate screening and counselling for appropriate interventions including low salt intake. The CKDNET has also provided the information about salt content in different food serving (https://ckd.kku.ac.th/food) as well as launched mobile applications named “H_2_O for life” and “CKD KKU”. These strategies are thought to help for prevention of CKD by facilitating salt and fluid self-monitoring.

#### Comprehensive CKD care

Comprehensive CKD care of the CKDNET is being provided by the formulation of local guidelines and creation of an alert list based on the patients’ EMR in the CKDNET registry. The guidance of primary care providers are extremely crucial in Thailand because the majority of CKD cases are screened and treated at primary and secondary care units. Therefore, CKDNET conducts annual meeting of local nephrologists, primary care physicians, nurses and pharmacists to share and deal the problems effectively, and facilitate widespread care. The situation of CKD in each region is reviewed and the future plans and guidelines are formulated at the meeting. Our alert list is designed to facilitate the treatment of CKD patients according to their clinical needs or severity.

#### Compliance improvement

We are applying an individualized care to help patients to achieve health and life goals. For this purpose, case manager nurses identify individual problems, apply the chronic care model & self-management supports, and integrate renal care by multidisciplinary approaches including primary care, pharmacy, nutrition, exercise, behavioral change, education and awareness. This procedure has been implicated in the primary hospitals. Every year the CKDNET conducts training for nurses to become a case manager nurses to deliver better care to CKD patients.

#### Cost-effectiveness analysis

The cost effectiveness of our proposed care models was analysed. The study involved three parts: (i) constructing CKD progression and complication model using the Markov model based on epidemiological data of Thai CKD patients and effectiveness data from the CKDNET; (ii) measuring the associated costs and EQ-5D-utility weight of patients with various CKD stages using primary data collection; and (iii) calculating the incremental cost-effectiveness ratio (ICER). The Markov model simulates the disease course of patients as they experience progressive CKD, ESRD, complications and death. The cost effectiveness of the CKDNET program was estimated by simulating the total lifetime costs and quality-adjusted life years (QALYs) for patients under the CKDNET program versus under conventional care. A wide array of sensitivity analyses with various assumptions were performed.

## Results

### CKD screening and identification of CKD factors

The screening program has been conducted in two rural sub-districts, Don Chang and Khok Samran of Khon Kaen Province covering 2789 persons, and 2205 of them have completed physical and chemical examinations twice. Our initial data revealed that the prevalence of CKD in these study areas was 26.8% of the screened population which was higher than that reported previously [9]**.** Hypertension (43.2%) and diabetes (35.8%) were the major CKD associated risk factors. Importantly, 28.9% of CKD patients or 7.76% of the total screened population and 12.9% of the population without risks of CKD were reported to have CKD of unknown causes. So far, we have achieved our goal of minimum number of screening of 1000 people in each of the two sub-districts.

### CKD registry

Currently, our CKD surveillance system shows that 225 hospitals are in the list and most of them are located in the Northeast Thailand. It displays that 10.4 million individuals are registered of which 1.73 million are confirmed to have major risk factors for CKD - diabetes and hypertension, and about 0.13 million are diagnosed as having CKD (data on December 22nd 2019). The target of CKD registry is to link all the 319 district and provincial hospitals in the Northeast Thailand to the Thai care cloud.

### Community-based CKD prevention

**Fig. 5:**
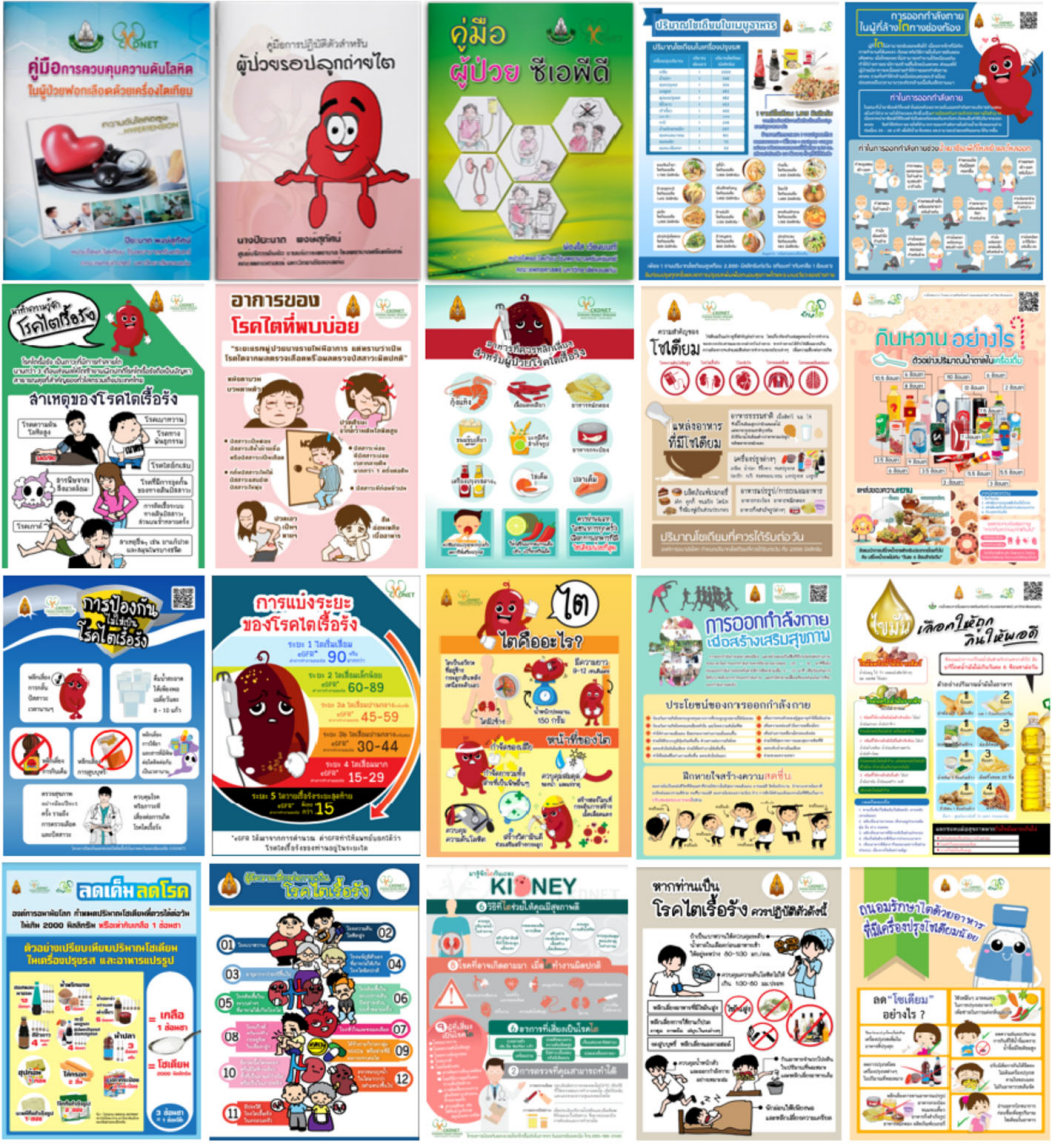
Representation of poster and pamphlets displaying various information for education and awareness against CKD

Pamphlets, posters, brochures, manuals, books, digital versatile discs and other medias of 94 different types in Thai language (Fig. 5) in the total number of 478,450 have been distributed for CKD education and awareness at the community level. These materials have reached to 90 health care units of 18 provinces in the region. Moreover, a preliminary study of measuring salt content in foods at six different canteens and a food center within KKU campus revealed that 72.7% (205/282 of food samples) had higher salt content than the healthy dishes (> 600 mg/dish), based on the WHO guideline that an adult should consumes < 2000 mg of sodium (equivalent to three dishes) per day (Table 2). Also, salt intake reduction campaign was conducted at 4 schools with 25 teachers and 199 students in Don Chang Sub-district. A course in the curriculum of primary students (grade 4) at KKU demonstration school was introduced as a pilot activity to improve knowledge on kidney diseases and its prevention in children. A total of 160 students were taught about correct nutrition, healthy habits and exercise, salt intake, reading nutrition label in packed food as well as kidney structure, kidney function, causes of kidney diseases, symptoms and prevention for 6 consecutive weeks (4 h/week) by faculty members of KKU. Most importantly, our CKD prevention strategies has been integrated into the Khon Kaen Province since 2018.

**Table 2:** Showing number of foods measured for sodium content before and after salt intake reduction campaign at canteens in KKU

Canteens in the Faculties	Number of dishes measured for sodium before campaign (%)			Number of dishes measured for sodium after campaign (%)		
	Meet the standard criteria	Over the standard criteria	Total	Meet the standard criteria	Over the standard criteria	Total
Medicine	33 (28.2)	84 (71.8)	117 (100)	27 (30.7)	61 (69.32)	88 (100)
Associated Medical Sciences	6 (28.6)	15 (71.4)	21 (100)	10 (55.6)	8 (44.4)	18 (100)
Pharmaceutical Sciences	3 (15.8)	16 (84.2)	19 (100)	6 (35.3)	11 (64.7)	17 (100)
Dentistry	7 (29.2)	17 (70.8)	24 (100)	11 (31.4)	24 (68.6)	35 (100)
Nursing	11 (40.7)	16 (59.3)	27 (100)	10 (47.6)	11 (52.4)	21 (100)
Veterinary Medicine	6 (33.3)	12 (66.7)	18 (100)	2 (15.4)	11 (84.6)	13 (100)
University Complex	11 (19.6)	45 (80.4)	56 (100)	42 (38.9)	66 (61.1)	108 (100)
Total	77 (27.3)	205 (72.7)	282 (100)	108 (36)	192 (64)	300 (100)

### Comprehensive CKD care

We have established the CKDNET guidelines for the treatment of CKD patients. These were formulated from the guidelines of KDIGO and the Nephrology Society of Thailand to match the characteristics of population, resources, and socio-economic aspects of the regions. Additionally, an alert list based on patients’ EMR in the CKDNET registry has been created for effectual comprehensive care. The list consists of 38 parameters (Table 3) that alerts the primary care physicians as well as nephrologists on single or cumulative abnormal conditions of a patient or hospital.

**Table 3:** Showing alert list in CKD registry

	Parameters/Conditions
1.	Systolic blood pressure > 130 mm of Hg
2.	Diastolic blood pressure > 90 mm of Hg
3.	Hemoglobin < 10 g/dl
4.	Not receiving angiotensin-converting-enzyme inhibitor (ACEI) and angiotensin II-receptor blocker (ARB)
5.	Slope of estimated glomerular filtration rate (eGFR) per period ≥4
6.	HbA1C < 6.5%
7.	HbA1C > 7.5%
8.	LDL cholesterol > 100 mg/dl
9.	Serum potassium > 5.5 mEq/L
10.	Serum bicarbonate < 22 mEq/L
11.	Not evaluated with urine protein strip
12.	Not evaluated for urine protein to creatinine ratio (UPCR) or urine protein 24 h
13.	UPCR ≥500 mg/g or Urine protein 24 h ≥ 500 mg /day
14.	Serum phosphate > 4.5 mg/L
15.	Serum parathyroid hormone (PTH) is not normal
16.	Did not receive AVF preparation before starting hemodialysis
17.	Participated in educational classes on various topics
18.	Notify this eGFR and CKD period and within the past 3 months
19.	eGFR value < 60 should see a doctor
20.	There is a decrease in the rate of GFR over 5 ml/min/1.73 m^2^ per year
21.	eGFR< 30 received a consultation on RRT
22.	eGFR< 30 also received metformin
23.	Diabetic patients with albuminuria > 30 mg/day and not receiving ACEI or ARB
24.	No diabetes, have albuminuria > 300 mg/day and do not receive ACEI or ARB
25.	Received ACEI/ARB, be aware of the occurrence of AKI and hyperkalemia. Advice should be given.
26.	Have received ACEI or ARB but had to stop the drug because of an adverse event
27.	Protein content in urine ≥1+
28.	Diabetics who do not check urine albumin at least 2 times a year
29.	Not being a CKD and receiving NSAIDs for more than 2 weeks
30.	CKD patients receiving NSAIDs
31.	Time for dispensing in the program if there is a drug that needs to be adjusted according to creatinine clearance (CrCl), if wrongly ordered, there will be a warning every time or if there is no adjustment according to CrCl, remind every time
32.	The history of diagnosing an acute kidney injury last day, month, year
33.	There is a disease or condition that is at risk of CKD, being tested for serum creatinine and urine protein or albumin once a year (diabetes, hypertension, gout, SLE, over 60 years old, receiving nephrotoxic drugs, upper urinary tract infection ≥3 times a year, with cardiovascular disease, polycystic kidney disease, kidney disease from birth, have a history of kidney disease in the family)
34.	UA detection of proteins or red blood cells in urine, consider sending a doctor
35.	Serum creatinine increases more than or equal to 0.3 mg/dl: acute kidney injury should determine the cause
36.	GFR < 30 ml/min/1.73m^2^ consider sending for consultation to a doctor
37.	GFR < 15 ml/min/1.73m^2^ should submit assessment for preparation for renal replacement therapy
38.	Being on ACEI/ARB and serum potassium > 5 mEq/L

### Compliance improvement

Our compliance improvement strategy using case-manager nurses showed high impact on management and care of CKD patients. In a randomized controlled trial with 200 participants aged 40–75 years in CKD stages II-IV, a case-manager nurse coordinated care with multi-disciplinary team of family doctor, dietician, pharmacist, physical therapist and others were implemented. They provided individual empowerment to 95 CKD patients in intervention group for 12 months. It was found that the intervention group appeared with significantly higher eGFR (49.57 versus 46.23 ml/min/1.73 m^2^; *p* < 0.05) and lower blood pressure (129.6/76.1 versus 135.8/83.6 mmHg) compared to the control group under conventional care [20]. Additionally, a statistically significant improvement of the self-management behavior mean score in intervention group (97.67 ± 9.14 versus 82.02 ± 7.01, *p* < 0.05) was noticed compared to CKD patients under conventional care. Therefore, we have expanded this program of self-management and case management by training 369 case-manager nurses in the last 2 years.

### Cost-effectiveness analysis

Since the CKDNET program has been proposed as a way to relieve the cost and morbidity associated with CKD in the Northeast Thailand, we analyzed the cost effectiveness of the proposed care models. It was assumed that over the five-year period, the CKDNET program would incur a total cost per patient of 1000 Baht per year. Costs of outpatient and inpatient services by CKD stages and complications are presented in the supplementary information. The early assessment of the CKDNET program on patient outcomes at the first year revealed that the program could slow the disease progression from stages 3 to 4 by 20% and stages 4 to 5 by 25%. The effect of the program on a decrease in complications including stroke, acute myocardial infarction, and congestive heart failure has not yet been available, thus a 20%-decrease in the risks was assumed. In the base case analysis, effectiveness of the program was assumed to persist for the first 5 years. The CKDNET program contributes a total of 11.22 QALYs, whereas the conventional care contributed a total of 10.82 QALYs. The lifetime cost for the comprehensive health services under the CKDNET program was 486,898 Baht and the cost of the conventional care was 479,386 Baht, resulting in an ICER of 18,702 Baht per QALY gained. If the annual cost of the CKDNET activities increased to 15,000 Baht per patient, the ICER would increase to 163,523 Baht/QALY, which was equal to the cost-effectiveness threshold in Thailand. The scenario analyses that varied assumptions on the program effectiveness confirmed that the program with an annual activity cost up to 6000 Baht per patient was still cost-effective even if the program could not decrease risks of disease complications. An additional file shows this in more detail (see Additional file 1).

## Discussion

The CKDNET is the first project of this kind in the Northeast Thailand. During a short period of 3 years since its establishment, the project has already reached a milestone in terms of CKD surveillance, while other activities are in progress. Our general rural population screening program revealed that one in four people in the region has CKD, which is a pathetic situation compared to 11–13% prevalence worldwide [2]. Although diabetes and hypertension were found to be the major CKD-associated risk factors in our study, CKD of unknown causes is also disturbing the local nephrologists by contributing nearly one third of the observed total CKD burden. Researches to elucidate the pathogenesis of unknown CKD are crucial and under investigation by our researchers. CKD screening activities promote public awareness and education, and serve as medical outreach to underserve populations [21, 22].

Moreover, the CKDNET hospital-based surveillance system provides a well-designed data for monitoring of population at risk of developing CKD, in particular, those who had diabetes and/or hypertension and once they developed CKD. This surveillance system hopes to facilitate research, professional development, and most importantly, health care/service improvement in Thailand. For instance, the number of patients at various stages of CKD and their status during the course of time can be tracked as well as survival estimates can be performed with ease. Therefore, we conduct annual training to physicians, nurses, and IT officers from all the public health sectors in the registry system. Through screening and surveillance, the CKDNET targets to screen at least 0.1 million population every year. One of such registry systems in Australia, CKD: QLD has brought about notable success to address the CKD problem [23].

Use of the pamphlets, posters and brochures for the awareness and prevention activities is an old but still successful methods. We used verities of contents including CKD, causes, risk groups, risk factors, salt content in common foods/drinks, diet and much more. We were aware that limited health literacy in rural areas may bound the usefulness of such written pamphlets. Nonetheless, our awareness materials are in use for the counselling of CKD patients in hospitals throughout the region. YouTube, Facebook, website, digital kiosk and mobile applications were also approached as modern methods and found resourceful for awareness and self-monitoring. We feel strongly motivated from the finding that mobile application assisted self-checking of CKD has satisfactory outcome in developing countries [24]. Our salt reduction campaign was effective to reduce the salt content in 8.7% of food menu prepared in the university canteens. Although the data are from small scale studies, still it gives a positive remark. It is well known that high salt intake is associated with hypertension and control of hypertension can reduce the risk of developing CKD [25].

Modification of the standard CKD treatment guidelines was one of our important strategy. Such modification is inevitable for comprehensive care because implementation of established guidelines for CKD in practice is challenging [26]. For example, in Thailand, nephrologist-to-patient ratio is extremely low. Our guideline instructed the treatment of stage 1 to 3 CKD patients at primary and secondary health care units by general physicians and nurses, while stage 4 to 5 CKD patients to be treated by the nephrologist at tertiary care units. It can thus reduce the constraint on nephrologist and co-ordinate the care.

Trained case-manager nurses for the self-management support is an effective method for the care and management of CKD patients. Our randomized cohort study at primary hospitals revealed that case managers can slower the CKD progression [20, 27]. However, we found it difficult at the beginning because of the shortage of the number of case-manager nurses. The situation is being dealt with the development and training of new case managers annually.

Next, an important part of our project was the cost effectiveness analysis of CKDNET care model compared to conventional CKD care in Thailand. The results show that our program is a best buy intervention because it not only increases QALYs but also slow down the progression of CKD to ESRD in about 25% of patients, hence avoids the need for dialysis or transplant. Furthermore, the ICER would touch the cost-effectiveness threshold if the annual cost of the CKDNET activities increased to 15,000 Baht per patient. We used Markov models for cost effective analysis as these are a type of decision-analysis model used to analyze uncertain processes, such as chronic kidney disease in which costs and outcomes occur over a long period of time [28].

The CKDNET project has wide-range of tasks to accomplish, among which, expansion of the project to entire nation is of prime importance. Moreover, the focus is in the CKDNET care model which is under several aspects of implementation throughout Don Chang Sub-district. Nutritional factors including environmental (water, air and soil) contaminants are on a list to be investigated for risk factors especially associated with CKDu. Discovery of biomarkers and sensors must be accelerated. Expansion and development of existing biobank is another central priority. In longer timespan, the CKDNET is collaborating with important stakeholders at national and international levels.

There are some limitations in our findings and strategies. Firstly, our CKD prevalence represents the status of Khon Kaen Province, and it is higher than the previously reported national data by Thai SEEK study. Therefore, the current incidence rate may not represent the true figure of all Thailand. Secondly, CKDNET registry is based on the hospital data so that the information from community level is missing. Thirdly, although we have trained nurse to work as a case manager, they were assigned with many other routine duties. Accordingly, we plan to provide training to CKD patients to empower other patients.

## Conclusions

The CKDNET project has outlined and instigated prolific strategies for prevention and management of CKD in the Northeast of Thailand. While many activities are in progress, CKD surveillance system that links hospitals to Thai health care platform has been successfully developed. We believe that our findings and endeavors will advance the efforts of clinicians, researchers and policy makers for a qualitative CKD care in the region.

## Supplementary information

**Additional file 1.** Information of all the various assumptions and price points used in the Markov modeling.

## Data Availability

The datasets used and/or analysed during the current study are available from the corresponding author on reasonable request.

## Abbreviations

CKD: Chronic kidney disease
CKDu: CKD of unknown origin
GFR: Glomerular filtration rate
QALYs: Quality-adjusted life years
ESRD: End stage renal disease
EMR: Electronic medical record
KDIGO: Kidney disease improving global outcomes
CKDNET: Chronic Kidney Disease Prevention in the Northeast of Thailand

## References

[1] GBD (2016). 2015 Mortality and Causes of Death Collaborators. Global, regional, and national life expectancy, all-cause mortality, and cause-specific mortality for 249 causes of death, 1980–2015: a systematic analysis for the Global Burden of Disease Study 2015. Lancet.

[2] Hill NR, Fatoba ST, Oke JL, Hirst JA, O'Callaghan CA, Lasserson DS (2016). Global prevalence of chronic kidney disease - a systematic review and meta-analysis. PLoS One.

[3] Whaley-Connell A, Shlipak MG, Inker LA, Kurella Tamura M, Bomback AS, Saab G (2012). Awareness of kidney disease and relationship to end-stage renal disease and mortality. Am J Med.

[4] Rao KSS, Kaptoge S, Thompson A, Di Angelantonio E, Gao P, Sarwar N (2011). Diabetes mellitus, fasting glucose, and risk of cause-specific death. N Engl J Med.

[5] Wilson PW, D'Agostino RB, Levy D, Belanger AM, Silbershatz H, Kannel WB (1998). Prediction of coronary heart disease using risk factor categories. Circulation..

[6] Jayatilake N, Mendis S, Maheepala P, Mehta FR (2013). Chronic kidney disease of uncertain aetiology: prevalence and causative factors in a developing country. BMC Nephrol.

[7] Tatapudi RR, Rentala S, Gullipalli P, Komarraju AL, Singh AK, Tatapudi VS (2019). High prevalence of CKD of unknown etiology in Uddanam, India. Kidney Int Rep.

[8] Torres C, Aragon A, Gonzalez M, Lopez I, Jakobsson K, Elinder CG (2010). Decreased kidney function of unknown cause in Nicaragua: a community-based survey. Am J Kidney Dis.

[9] Ingsathit A, Thakkinstian A, Chaiprasert A, Sangthawan P, Gojaseni P, Kiattisunthorn K (2010). Prevalence and risk factors of chronic kidney disease in the Thai adult population: Thai SEEK study. Nephrol Dial Transplant.

[10] Ong-Ajyooth L, Vareesangthip K, Khonputsa P, Aekplakorn W (2009). Prevalence of chronic kidney disease in Thai adults: a national health survey. BMC Nephrol.

[11] Yanagawa M, Kawamura J, Onishi T, Soga N, Kameda K, Sriboonlue P (1997). Incidence of urolithiasis in Northeast Thailand. Int J Urol.

[12] Hemmelgarn BR, Zhang J, Manns BJ, James MT, Quinn RR, Ravani P (2010). Nephrology visits and health care resource use before and after reporting estimated glomerular filtration rate. JAMA..

[13] Plantinga LC, Boulware LE, Coresh J, Stevens LA, Miller ER, Saran R (2008). Patient awareness of chronic kidney disease: trends and predictors. Arch Intern Med.

[14] Chronic Kidney Disease Prevention in the Northeast of Thailand. Available from: https://ckd.kku.ac.th/ [Accessed 26.12.2019].

[15] Barrett BJ, Garg AX, Goeree R, Levin A, Molzahn A, Rigatto C (2011). A nurse-coordinated model of care versus usual care for stage 3/4 chronic kidney disease in the community: a randomized controlled trial. Clin J Am Soc Nephrol.

[16] Devins GM, Mendelssohn DC, Barre PE, Binik YM (2003). Predialysis psychoeducational intervention and coping styles influence time to dialysis in chronic kidney disease. Am J Kidney Dis.

[17] Saran R, Hedgeman E, Plantinga L, Burrows NR, Gillespie BW, Young EW (2010). Establishing a national chronic kidney disease surveillance system for the United States. Clin J Am Soc Nephrol.

[18] Trivedi HS, Pang MM, Campbell A, Saab P (2002). Slowing the progression of chronic renal failure: economic benefits and patients’ perspectives. Am J Kidney Dis.

[19] KDIGO CKD Work Group (2013). KDIGO 2012 clinical practice guideline for the evaluation and Management of Chronic Kidney Disease. Kidney Int Suppl.

[20] Kankarn W, Tongkrajai P, Kumphon B, Anutrakulchai S (2019). The impact of self-management and case management on progression of chronic kidney disease in urban communities of Khon Kaen. J Med Assoc Thail.

[21] Brown WW, Peters RM, Ohmit SE, Keane WF, Collins A, Chen SC (2003). Early detection of kidney disease in community settings: the kidney early evaluation program (KEEP). Am J Kidney Dis.

[22] Wald NJ (2001). The definition of screening. J Med Screen.

[23] Venuthurupalli SK, Hoy WE, Healy HG, Salisbury A, Fassett RG, CKD (2012). QLD: chronic kidney disease surveillance and research in Queensland, Australia. Nephrol Dial Transplant.

[24] Sobrinho A, da Silva LD, Perkusich A, Pinheiro ME, Cunha P (2018). Design and evaluation of a mobile application to assist the self-monitoring of the chronic kidney disease in developing countries. BMC Med Inform Decis Mak.

[25] Vollmer WM, Sacks FM, Ard J, Appel LJ, Bray GA, Simons-Morton DG (2001). Effects of diet and sodium intake on blood pressure: subgroup analysis of the DASH-sodium trial. Ann Intern Med.

[26] Fox CH, Voleti V, Khan LS, Murray B, Vassalotti J (2008). A quick guide to evidence-based chronic kidney disease care for the primary care physician. Postgrad Med.

[27] Kankarn W, Wichitthongchai C, Sancharon P, Anutrakulchai S (2019). The effects of individualized nutritional management to slow the progression of stage 3 to 4 chronic kidney disease in primary care units of Northeast Thailand. J Med Assoc Thail.

[28] Gray AM, Clarke PM, Wolstenholme JL, Wordsworth S (2011). Applied methods of cost-effectiveness analysis in healthcare.

