# Supplementary material for: CKDNET, a quality improvement project for prevention and reduction of chronic kidney disease in the Northeast Thailand
Source: BMC Public Health. 2020 Aug 27;20:1299. doi: 10.1186/s12889-020-09387-w (PMC7450931; doi:10.1186/s12889-020-09387-w)
Supplement: Supplementary file 1 — Additional file 1. Information of all the various assumptions and price points used in the Markov modeling. [file 12889_2020_9387_MOESM1_ESM.docx]

**Table S1.** Direct health care cost for patients with CKD in various stages and clinical conditions

| Cost item | Value  (Baht per year) | Data source |
| --- | --- | --- |
| Outpatient care |  |  |
| CKD stage 1 | 1,900 | Outpatient data, Khon Kaen Hospital |
| CKD stage 2 | 2,779 |  |
| CKD stage 3 | 16,168 | Pooled estimate of outpatient data from 3 hospitals (Srinagarind Hospital, Khon Kaen Hospital, and Maha Sarakham Hospital) |
| CKD stage 4 | 17,745 |  |
| CKD stage 5 | 27,158 |  |
| Inpatient care |  |  |
| CKD stage 1 | 6,114 | Outpatient and inpatient data, Khon Kaen Hospital |
| CKD stage 2 | 6,114 |  |
| CKD stage 3 | 13,385 | Pooled estimate of inpatient data from 3 hospitals (Srinagarind Hospital, Khon Kaen Hospital, and Maha Sarakham Hospital) |
| CKD stage 4 | 14,718 |  |
| CKD stage 5 | 14,830 |  |
| Outpatient and inpatient care |  |  |
| CKD with peritoneal dialysis, year 1 | 473,191 | Permsuwan et al. 2016 |
| CKD with peritoneal dialysis, year 2 onwards | 419,665 |  |
| CKD with hemodialysis, year 1 | 464,955 |  |
| CKD with hemodialysis, year 2 onwards | 440,295 |  |
| Congestive heart failure, year 1 | 60,546 |  |
| Congestive heart failure, year 2 onwards | 26,175 |  |
| Stroke, year 1 | 75,010 | Tamteeranon Y. 2008 |
| Stroke, year 2 onwards | 11,883 |  |
| Myocardial infarction, year 1 | 152,103 |  |
| Myocardial infarction, year 2 onwards | 16,265 |  |

CKD: chronic kidney disease, THB: Thai Baht

Permsuwan U, Chaiyakunapruk N, Dilokthornsakul P, Thavorn K, Saokaew S. Long-Term Cost-Effectiveness of Insulin Glargine Versus Neutral Protamine Hagedorn Insulin for Type 2 Diabetes in Thailand. Appl Health Econ Health Policy. 2016;14(3):281-292.

Tamteeranon Y. Cost utility analysis of HMG Co-A reductase inhibitors (statins) for primary prevention on cardiovascular disease (Thesis). Mahidol University; 2008.

**Table S2.** Quality-adjusted life years, lifetime costs, incremental cost-effectiveness ratios and activity costs by annual cost per person

| Option | QALYs | Annual cost of CKDNET program per person (Baht) | | | | | | | |
| --- | --- | --- | --- | --- | --- | --- | --- | --- | --- |
|  |  | 1,000 | 2,000 | 3,000 | 4,000 | 5,000 | 6,000 | 10,000 | 20,000 |
| a. No CKDNET program | 10.82 | 479,386 | 479,386 | 479,386 | 479,386 | 479,386 | 479,386 | 479,386 | 479,386 |
| Scenario 1 |  |  |  |  |  |  |  |  |  |
| b. With CKDNET program | 11.22 | 486,898 | 491,052 | 495,207 | 499,362 | 503,517 | 507,672 | 524,291 | 565840 |
| Difference (b - a) | 0.40 | 7,512 | 11,666 | 15,821 | 19,976 | 24,131 | 28,286 | 44,905 | 86,454 |
| ICER (Baht/QALY) | - | 18,702***** | 29,046 | 39,391 | 49,735 | 60,079 | 70,424 | 111,801 | 215,245 |
| Scenario 2 |  |  |  |  |  |  |  |  |  |
| c. With CKDNET program | 12.06 | 525,490 | 536,665 | 547,840 | 559,015 | 570,190 | 581,365 | 626,064 | 737,813 |
| Difference (c - a) | 1.24 | 46,104 | 57,279 | 68,454 | 79,629 | 90,804 | 101,979 | 146,678 | 258,427 |
| ICER (Baht/QALY) | - | 37,249 | 46,278 | 55,306 | 64,335 | 73,363 | 82,392 | 118,506 | 208,791 |
| Scenario 3 |  |  |  |  |  |  |  |  |  |
| d. With CKDNET program | 11.00 | 487,275 | 491,397 | 495,519 | 499,640 | 503,762 | 507,883 | 524,370 | 565,586 |
| Difference (d - a) | 0.18 | 7,889 | 12,011 | 16,133 | 20,254 | 24,376 | 28,497 | 44,984 | 86,200 |
| ICER (Baht/QALY) | - | 44,350 | 67,520 | 90,689 | 113,859 | 137,028 | 160,197 | 252,875 | 484,570 |

QALYs: quality-adjusted life years; ICER: incremental cost-effectiveness ratio

*Base-case analysis

**Scenario 1:** Five-year activities of CKDNET program that resulted for the first five years in an annual decrease in CKD progression from stages 3 to 4 and from stages 4 to 5, respectively, by 20% and by 25% plus an annual decrease in the risks of stroke, acute myocardial infarction, and congestive heart failure by 20%.

**Scenario 2:** Continual activities of CKDNET program that resulted in a lifetime, annual decrease in CKD progression from stages 3 to 4 and from stages 4 to 5, respectively by 20% and by 25%. The program did not decrease the risks of stroke, acute myocardial infarction, and congestive heart failure.

**Scenario 3:** Five-year activities of CKDNET program that resulted for the first five years in an annual decrease in CKD progression from stages 3 to 4 and from stages 4 to 5, respectively, by 20% and by 25%. The program did not decrease the risks of stroke, acute myocardial infarction, and congestive heart failure.
